# Medication and Outcome in Older Heart Failure Patients: Results from a Prospective Cohort Study

**DOI:** 10.3390/ph17060711

**Published:** 2024-05-30

**Authors:** David Peter Garay, Hugo Saner, Jan Herzberg, Gerrit Hellige, Nisha Arenja

**Affiliations:** 1 Department of Cardiology, Kantonsspital Olten and Bürgerspital Solothurn, Solothurner Spitäler AG, 4500 Solothurn, Switzerland; david.garay@spital.so.ch (D.P.G.); jan.herzberg@spital.so.ch (J.H.); gerrit.hellige@spital.so.ch (G.H.); 2 Institute for Social and Preventive Medicine and ARTORG Center for Biomedical Engineering Research, University of Bern, 3012 Bern, Switzerland; hugo.saner@bluewin.ch; 3 Faculty of Medicine, University of Basel, 4001 Basel, Switzerland

**Keywords:** heart failure, older patients, medical treatment, prognosis, rehospitalization, mortality

## Abstract

**Purpose**: Acute heart failure (AHF) is associated with high morbidity and mortality, and the prognosis is particularly poor in older patients. Although the application of guideline-directed medical therapy (GDMT) has shown a positive impact on prognosis, the effects are less clear in older age groups. The aim of this study was to analyze real-world data regarding GDMT and outcomes in older HF patients. **Methods**: This is a prospective cohort study from a secondary care hospital in central Switzerland. A total of 97 consecutive patients aged ≥60 years were enrolled between January 2019 and 2022. The main outcome parameters were prescribed GDMT at discharge, and in case of rehospitalization, GDMT at readmission, and survival in terms of all-cause mortality and HF-related hospitalizations during a 3-year follow-up period. **Results**: Follow-up data were available for 93/97 patients. The mean age was 77.8 ± 9.8 years, 46% being female. The mean left ventricular ejection fraction (LVEF) was 35.3 ± 13.9%, with a mean BNP level of 2204.3 ± 239 ng/L. Upon discharge, 86% received beta-blockers and 76.3% received renin–angiotensin system (RAS) inhibitors. At rehospitalization for AHF, beta-blockers use was significantly lower and decreased to 52.8% (*p* = 0.003), whereas RAS inhibitor use increased slightly to 88.9% (*p* = 0.07), and SGLT-2 inhibitors showed a significant increase from 5.4% vs. 47.2% (*p* = 0.04). GDMT prescription was not dependent on LVEF. Overall, 73.1% of patients received two-stage or three-stage GDMT at discharge, whereas this percentage decreased to 61% at rehospitalization (*p* = 0.01). Kaplan–Meier analysis for the combined outcome rehospitalization and death stratified by LV function showed significant differences between LVEF groups (aHR: 0.6 [95% CI: 0.44 to 0.8]; *p* = 0.0023). **Conclusions**: Our results indicate that first, the majority of older AHF patients from a secondary care hospital in Switzerland were not on optimal GDMT at discharge and even fewer at readmission, and second, that prognosis of the population is still poor, with almost half of the patients having been rehospitalized or died during a 3-year follow-up period under real-world conditions, without significant difference between women and men. Our findings underline the need for further improvements in the medical treatment of AHF, in particular in older patients, to improve prognosis and to reduce the burden of disease.

## 1. Introduction

Heart failure (HF) is associated with high mortality, low functional status and poor quality of life (QOL). In the general population, the prevalence of HF is 2% and increases to >10% in patients over 70 years of age [1,2,3,4,5]. Furthermore, acute HF (AHF) is the most common cause of hospitalization in patients >65 years of age [6]. With each hospitalization, reintegration into familiar living conditions becomes more difficult in this age group and is associated with a decrease in QOL and a high economic and social burden [7].

Advances in pharmacotherapy for HF have led to considerable improvements in mortality and morbidity over the last ten years [8]. Currently, guideline-directed medical therapy (GDMT) is the mainstay of HF treatment in patients with reduced ejection fraction (HFrEF) [9,10]. GDMT includes four main categories: beta-blockers, renin–angiotensin system (RAS) inhibitors, mineralocorticoid receptor antagonists (MRAs), and sodium-glucose cotransporter-2 inhibitors (SGLT-2 inhibitors).

However, there is insufficient evidence regarding the use and effectiveness of these treatments in older patients with HFrEF. Patients aged over 60 years are underrepresented or even excluded in large controlled clinical trials [11]. As a result, opportunities to translate results from such studies to the treatment of older patient in the general population are limited.

Therefore, the aim of this study was to analyze real-world data from a secondary care center in central Switzerland in regard to GDMT prescription and outcomes in a cohort of consecutive older patients hospitalized for AHF.

## 2. Results

### 2.1. Patients Characteristics

Overall, 93/97 patients from the registry could be included into the final analysis with complete data. Baseline characteristics of the study population are shown in Table 1. At the first hospitalization for HF decompensation, patients were hospitalized for an average of 9 days. A total of 27/93 (29%) had an ischemic cause and 66/93 (71%) a non-ischemic cause and for HF decompensation. Non-ischemic causes of HF decompensation included severe aortic stenosis 4/93 (4.3%), transthyretin amyloidosis 1/93 (1.1%), cor pulmonale 1/93 (1.1%), dilated cardiomyopathy 18/93 (19.4%), hypertrophic cardiomyopathy 1/93 (1.1%), hypertensive heart disease 24/93 (25.8%), moderate to severe mitral regurgitation 4/93 (4.3%), takotsubo cardiomyopathy 3/93 (3.2%), atrial flutter 1/93 (1.1%), and atrial fibrillation 9/93 (9.7%).

A total of 28/93 (30%) had a first diagnosis of acute decompensated HF at presentation, while 65/93 (70%) were hospitalized with acute decompensation for known chronic HF (CHF). Overall, 33/93 (35%) had LVEF <30%, 26/93 (28%) LVEF 30–40%, and 34/63 (37%) LVEF 41–49%. The mean age of the population was 77.8 ± 9.8 years. BMI scores ranged from 22.3–30.8 kg/m^2^ (27.2 ± 6.6). The majority of patients were in NYHA functional class II and III, and the mean BNP level was 2204.3 ± 239 ng/L. Except for renal function (138.5 ± 85.4) and creatinine levels (102.9 ± 39.9 μmol/L in women vs. 169 ± 101.4 μmol/L in men, *p* < 0.001), there were no significant differences in baseline characteristics between women and men (Table 1).

### 2.2. Heart Failure Medication

**Figure 1:**
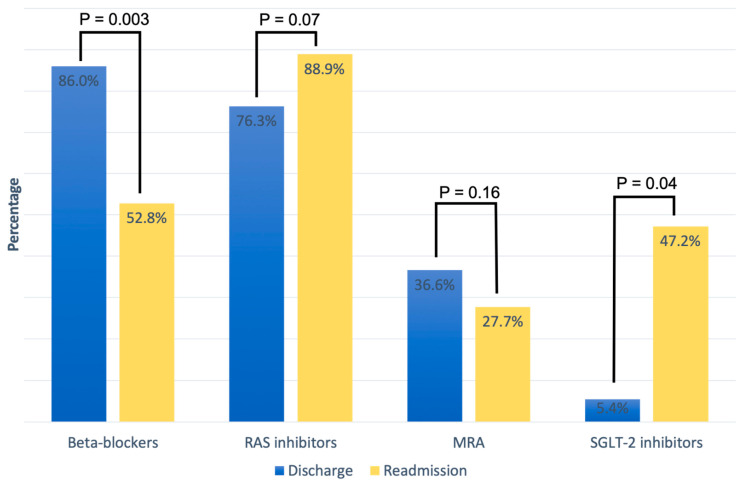
Medication for heart failure treatment at discharge and at readmission.

Table 2 summarizes the cardiovascular medical therapy upon discharge and readmission based on LV function categories. At discharge from the hospital, GLMT included the following medications: 86% beta-blockers, 76.3% RAS inhibitors, 36.6% MRAs, and 5.4% SGLT-2 inhibitors. Overall, there was no significant difference in cardiovascular medication at hospital discharge between the three LV function categories. A direct comparison of HF medication between discharge and readmission in patients readmitted for AHF showed a non-significant increase in treatment with RAS inhibitors (76.3% vs. 88.9%, *p* = 0.07) and a significant of SGLT-2 inhibitors (5.4% vs. 47.2%; *p* = 0.04), while the proportion of patients on beta-blockers (86% vs. 52.8%; *p* = 0.003) decreased significantly. MRA treatment also decreased on readmission compared to discharge, but without a significant difference (36.6% vs. 27.7%, *p* = 0.16) (Figure 1).

At readmission, patients with severely reduced LV function were significantly more often treated with an angiotensin receptor/neprilysin inhibitor (ARNI) (LVEF < 30%: 40% vs. LVEF > 41%: 11%, *p* = 0.04), There were no significant differences regarding other cardiovascular medications between the LV function categories (Table 2). 

**Figure 2:**
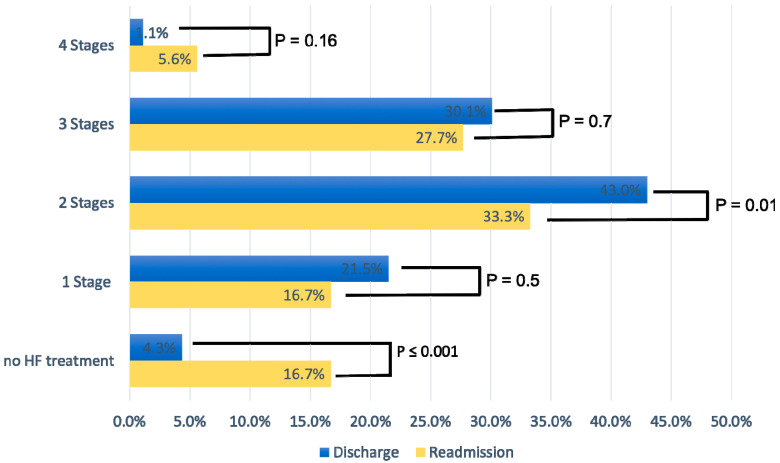
Medication for heart failure treatment according to four different treatment stages.

Figure 2 displays the percentage of patients according to the four stages of GDMT at discharge and readmission. At discharge, 43% of patients were on a two-stage GDMT and 30.1% on a three-stage GDMT. At readmission, the percentage of patients at all stages of GDMT (one to three) was considerably lower compared to hospital discharge; the difference was statistically significant for three-stage GDMT (*p* = 0.01). In addition, the percentage of patients who did not receive HF therapy at readmission increased significantly (4.3% vs. 16.7%, *p* < 0.001), with non-compliance with therapy being the main reason.

**Figure 3:**
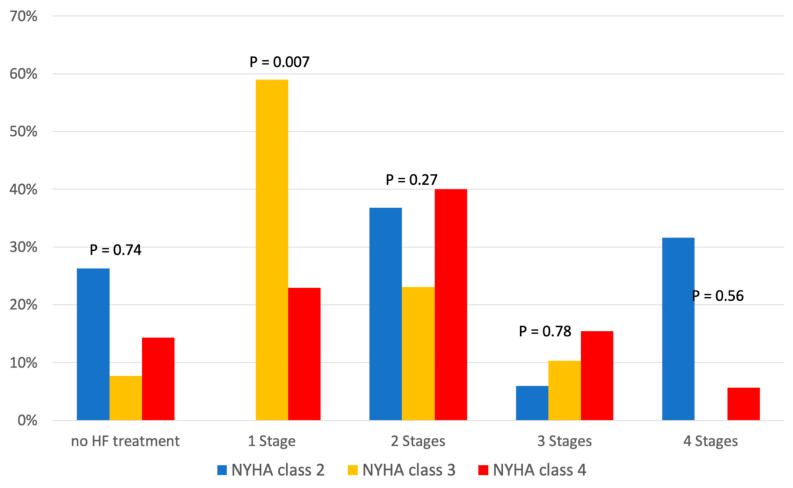
Medication for heart failure treatment according to New York Heart Association (NYHA) classes.

Additionally, Table 3 shows the relationship between HF medication and NYHA class, and Figure 3 shows its distribution according to the four stages of GDMT. We found no significant difference in HF therapy in relation to NYHA class (Table 3). While NYHA class 3 patients most frequently received one-stage HF therapy (59%), the proportion of NYHA class 4 patients receiving two-stage HF therapy increased to 40%, without any significant difference between discharge and readmission (Figure 3). The influence of comorbidities on treatment is shown in Table 4.

### 2.3. Outcomes

In the total cohort, 39% of the patients were readmitted due to decompensated AHF, with a median rehospitalization length of 7 days. The Cox regression analysis showed a significantly higher rate of rehospitalization for decompensated HF in patients with severely reduced LV function (adjusted hazard ratio (aHR): 0.52 [95% CI: 0.32 to 0.85]; *p* = 0.009). Death from all causes occurred in 47% of the study population during a 3-year follow-up. The combined outcome including rehospitalization for decompensated AHF and all-cause mortality occurred in 76.3% of the study population. A Kaplan–Meier analysis according to LVEF categories for the combined outcome showed a significant difference between groups (aHR: 0.6 [95% CI: 0.44 to 0.8]; *p* = 0.0023). We found no significant difference in combined endpoint among patients with and without beta-blockers treatment at readmission (*p* = 0.76).

## 3. Discussion

Our results indicate that first, the majority of older AHF patients from a secondary care hospital in Switzerland were not on optimal GDMT at discharge and even fewer patients at readmission, and second, that prognosis is still poor with almost half of the patients having been rehospitalized or died during a 3-year follow-up period under real-world conditions, with no significant difference between women and men. At hospital admission for decompensated AHF, LVEF was moderately reduced in most patients with an average LVEF of 35.3%. As expected, LVEF was an independent predictor for rehospitalization and mortality. Most patients were treated with beta-blockers and RAS inhibitors at hospital discharge, whereas almost half of the population received only two-stage GDMT (43%). During the follow-up period until rehospitalization, the number of patients on beta-blockers decreased, whereas the number of patients with RAS increased. The study by Lai HY et al., which was conducted in a patient population like ours, came to a similar conclusion. The majority of their patients were treated with beta-blockers and RAS inhibitors, as well as diuretics. They also showed that HF medication was reduced after discharge [12]. Another study by Qin X et al. showed that the discontinuation of RAS inhibitors and beta-blockers after discharge was common and the long-term adherence to HF medications was suboptimal [13]. The most common reasons for discontinuation of beta-blockers after discharge in our study population were hypotension and bradycardia. However, there might be a variety of additional reasons including such as worsening of chronic obstructive pulmonary disease and sick sinus syndrome [14]. A promising finding is that the percentage of patients with SGLT-2 inhibitors increased from 5.4% to 47.2%, which indicates a relatively fast adoption of new medications after inclusion into GDMT. 

The decision-making process regarding the initiation and continuation of drug treatment for HFrEF during hospitalization is complex and often depends on multiple factors such as the patient’s comorbidities and adherence and the discretion of the treating physician [15]. First, comorbidities often impede the upward dosing of established treatments, as many patients experience increased side effects and poorer tolerability. This is particularly true in frail individuals who may have increased intolerance, especially to antihypertensive medications [15,16,17]. Additionally, certain therapies may not be feasible due to disease-related limitations such as impaired renal function. Economic considerations also play an important role, though possibly to a lesser extent in a high-income country like Switzerland, where the cost of medications is fully covered by the health insurance. 

New therapeutic options that have been shown to be effective for the treatment of HF in large-scale studies, such as SGLT-2 inhibitors, are still underused [18,19]. Our results showed that 5.4% of patients received SGLT-2 inhibitors at discharge with a significant increase to 47.2% at readmission for AHF. In contrast, long-established HF-GDMT such as RAS inhibitors and beta-blockers are generally more frequently described at discharge [20]. Nevertheless, only 30% of HF patients were on a three-stage therapy, and only 1% received a four-stage HF-GDMT regime. Given the limited data from randomized studies for GDMT in older patients, observational evidence must serve as the primary source of knowledge to assume a similar treatment efficacy in older patients with HFrEF as that in younger patients [17,21,22]. For example, Barry et al. examined the use of GDMT in octogenarian and nonagenarian patients and found that the three main components of GDMT have only been prescribed in 25% of their patients [22]. 

Overall, our data demonstrate a high rate of rehospitalizations and a high mortality among older AHF patients with reduced LVEF, both being comparable to larger prospective cohort studies and register data [23,24,25]. Although we cannot directly link poor outcomes with inappropriate drug therapy, it is evident that appropriate use of GDMT should be considered for patients of all ages, including older HF patients [9,10,26]. In addition, most of the population was readmitted due to acute coronary syndrome and hypertensive heart disease. The lack of an interventional procedure could be another explanation for the high readmission rate. Furthermore, blood pressure control was not adequate in several patients with elevated blood pressure.

This poses a particular challenge for the healthcare system on a global level, which has to cope with an increasing number of older patients and needs to develop strategies to improve medical care for this age group [27]. It has been suggested that specialized centers with trained staff should be established to reduce the burden of early rehospitalization [13]. However, as most patients attend primary and secondary care centers, established GDMT should be the cornerstone of HF treatment under any circumstances, and efforts should be made to achieve target doses for GDMT, with vigilant monitoring for potential adverse drug events. However, it must be kept in mind that the pharmacokinetic characteristics of GDMT may be different in older compared to younger patients, with older patients potentially facing increased risks of adverse events. In this regard, our results add to current knowledge about problems and limitations of GDMT in older HF patients.

## 4. Material and Methods

### 4.1. Study Design and Population

This is a prospective cohort study in a secondary care hospital in central Switzerland representing conditions for the majority of HF patients in Switzerland. We enrolled 97 consecutive patients aged ≥60 years who experienced AHF and were admitted to our hospital between January 2019 and 2021. Diagnosis of AHF and medical interventions followed the current guidelines of the European Society of Cardiology (ESC) [9,10]. Data on the four main HF medications (beta-blockers, RAS inhibitors, MRAs, and SGLT-2 inhibitors) were collected for evaluation of GDMT. Each medication was recorded as one stage adding up to a maximum of four stages. We used the following inclusion criteria: (1) age ≥ 60 years, (2) hospitalization for acute decompensated HFrEF and left ventricular ejection fraction (LVEF) < 50%, (3) New York Heart Association (NYHA) class II to IV, and (4) the provision of informed consent. Exclusion criteria were (1) severe neuromuscular or oncological diseases with a predictably poor prognosis, (2) recent use of inotropic intravenous agents (≤10 days), (3) unstable angina, (4) severe uncontrolled arrhythmias, and (5) significant pulmonary limitations (Tiffeneau ratio < 70%, or vital capacity < 70% of predicted value).

The set-up of the registry for this study and the use of the data including data protection measures have been approved by the local ethics committee. 

### 4.2. Study Outcomes

We collected data on patient demographics, clinical characteristics, NYHA functional class, vital parameters, ECG, laboratory data, echocardiographic data, and device treatment at hospital entry. At hospital discharge, patients were evaluated for cardiac recompensation. In addition, HF medication has again been recorded at hospital entry after readmission. The primary outcome included the changes in GDMT for HF between the first hospitalization and readmission for decompensated HF. Secondary outcomes included (1) HF-related hospital readmission and all-cause mortality and (2) composite endpoint of all-cause mortality and HF-related hospital readmission over a follow-up period of 3 years. HF readmission was defined as any new non-elective inpatient HF hospitalization. Overall mortality was determined based on death data from the medical records and by contacting relatives. Outcome data were categorized into three groups according to LV function: (1) mildly reduced (LVEF 41–49%), (2) moderately reduced (LVEF 30% to 40%), and (3) severely reduced (LVEF < 30%). 

### 4.3. Statistics

Categorical variables are given as number and percentage, continuous parametric variables as mean ± standard deviation (SD). For the comparison of means between groups two-tailed Student’s *t*-test was used and differences between nominal variables were assessed using the Fisher exact test. Proportions of categorical were compared using Chi-squared test. Kaplan–Meier curves were used to estimate the distribution of survival as a function of the follow-up duration. Optimal cut-off values were defined by Receiver operating characteristics (ROC) and Youden’s J statistic. Differences were considered statistically significant at *p* < 0.05. Statistical data analyses were calculated using MedCalc 15 (MedCalc™, Mariakerke, Belgium).

## 5. Limitations

The major study limitation is the low number of participants that could be included in the study. However, the study population includes all patients > 60 years who were hospitalized in our secondary care hospital over a two-year period, and there is no obvious reason to assume that the situation is different in other secondary care hospitals with a similar service area or that the results could be significantly different by continuing patient recruitment over a longer time period. A further limitation is that we do not have data on medication adherence from our patients. Therefore, a potentially significant impact factor for the course of disease is missing. Finally, the SARS-CoV-2 pandemic may have influenced hospital admissions, length of stay, and outcomes, but it did not impact data collection or analysis.

## 6. Conclusions

Our results indicate that despite high rates of rehospitalization and poor prognosis, patients with AHF are still inadequately treated, both at hospital discharge and to an even greater extent during the follow-up period until rehospitalization. This applies to both women and men. Almost half of the patients were rehospitalized or died during a 3-year follow-up. Our findings underline the need for further improvements in the medical treatment of AHF particular in older patients to improve their prognosis and reduce the burden of disease.

## Figures and Tables

**Table 1:** Baseline characteristics of 93 study patients with hospitalization for decompensated heart failure.

	All (n = 93)	Women (n = 43)	Men (n = 50)	*p*-Value
Demographics, median (IQR)				
Age (years)	77.8 ± 9.8	79.1 ± 9.5	76.7 ± 10	0.74
BMI (kg/m^2^)	27.2 ± 6.6	26.4 ± 6.5	27.8 ± 6.8	0.82
Days of hospitalization	10.6 ± 6.7	10.8 ± 5	10.5 ± 7.9	0.3
NYHA functional class, n (%)				
II	19 (20.4)	8 (18.6)	11 (22)	0.16
III	39 (41.9)	20 (46.5)	19 (38)	0.68
IV	35 (37.6)	15 (34.9)	20 (40)	0.26
Vital parameter, median (IQR)				
Systolic blood pressure (mmHg)	134.5 ± 30.1	135.6 ± 26.7	133.5 ± 33	0.16
Diastolic blood pressure (mmHg)	82.4 ± 21.6	82.3 ± 20	82.6 ± 23.1	0.34
Heart rate at rest (bpm)	86.7 ± 19.3	88.7 ± 18.8	84.9 ± 19.7	0.76
ECG at baseline, n (%)				
Sinus rhythm	57 (61.3)	23 (53.5)	34 (68)	0.146
Atrial fibrillation	32 (34.4)	18 (41.9)	14 (28)	0.143
Paced rhythm	4 (4.3)	2 (4.7)	2 (4)	0.016
Laboratory data, median (IQR)				
BNP (ng/L)	2204.3 ± 239	2231.1 ± 258	2180.8 ± 225	0.42
Creatinine (μmol/L)	138.5 ± 85.4	102.9 ± 39.9	169 ± 101.4	<0.001
HbA1c (%)	5.4 ± 2.6	4.7 ± 2.9	6 ± 2.2	0.09
Echo Data, median (IQR)				
LVEF (%)	35.3 ± 13.9	34.6 ± 12.2	35.8 ± 15.3	0.14
LVEF groups, n (%)				
LVEF 41–50%:	34 (36.6)	16 (37.2)	18 (36)	0.9
LVEF 30–40%	26 (28)	13 (30.2)	13 (26)	0.65
LVEF < 30%:	33 (35.5)	14 (32.6)	19 (38)	0.59
Device treatment, n (%)				
Pacemaker	15 (16.1%)	6 (14)	9 (18)	0.6
ICD	5 (5.4%)	1 (2.3)	4 (8)	0.124

Abbreviations: BMI, body mass index; BNP, brain natriuretic peptide; ICD, implantable cardioverter defibrillator; LVEF, left ventricular ejection fraction; NYHA, New York Heart Association.

**Table 2:** Discharge (n = 93) and readmission (n = 36) medication according to left ventricular ejection fraction.

	Discharge				Readmission			
	LVEF <30% (n = 33)	LVEF 30–40% (n = 26)	LVEF 41–49% (n = 34)	*p*-Value	LVEF <30% (n = 10)	LVEF 30–40% (n = 8)	LVEF 41–50% (n = 18)	*p*-Value
Beta-blockers	27 (81.8)	22 (84.6)	31 (91.1)	0.53	4 (40)	5 (62.5)	10 (55.6)	0.91
Angiotensin-converting enzyme (ACE) inhibitors	14 (42.4)	8 (30.8)	12 (35.3)	0.64	2 (20)	5 (62.5)	10 (55.6)	0.17
Angiotensin II receptor blockers (ARBs)	3 (9.1)	6 (23.1)	3 (8.8)	0.19	1 (10)	2 (25)	6 (33.3)	0.54
Angiotensin receptor/neprilysin inhibitor (ARNI)	7 (21.2)	5 (19.2)	13 (38.2)	0.17	4 (40)	0	2 (11.1)	0.04
ARNI/ACEI/ARB	24 (72.7)	19 (73.1)	28 (82.4)	0.42	7 (70)	7 (84.5)	18 (100)	0.06
Mineralocorticoid receptor antagonist (MRA)	9 (27.3)	9 (34.6)	16 (47.1)	0.24	5 (50)	0	5 (27.8)	0.16
Sodium-glucose cotransporter-2 (SGLT2) inhibitors	0	3 (11.5)	2 (5.9)	0.147	4 (40)	6 (75)	7 (38.9)	0.37
Aspirin	7 (21.2)	8 (30.8)	5 (14.7)	0.32	1 (10)	1 (12.5)	3 (16.7)	0.78
Direct factor X anticoagulation	20 (60.6)	15 (57.7)	20 (58.8)	0.97	7 (70)	5 (62.5)	9 (50)	0.058
Vitamin K Antagonist	3 (9.1)	1 (3.8)	4 (11.8)	0.55	0	0	5 (27.8)	0.04
Calcium antagonists	5 (15.2)	2 (7.7)	2 (5.9)	0.4	1 (10)	1 (12.5)	1 (5.6)	0.51
Loop diuretics	20 (60.6)	22 (84.6)	25 (73.5)	0.12	6 (60)	5 (62.5)	16 (88.9)	0.32

**Table 3:** Heart failure medication according to New York Heart Association (NYHA) classes.

	NYHA Class 1 (n = 19)	NYHA Class 2 (n = 39)	NYHA Class 3 (n = 35)	*p*-Value
Beta-blockers	14 (73.7)	26 (66.7)	22 (62.9)	0.65
Angiotensin-converting enzyme (ACE) inhibitors	5 (26.3)	16 (41)	8 (22.9)	0.21
Angiotensin II receptor blockers (ARBs)	1 (5.3)	7 (18)	11 (31.4)	0.07
Angiotensin receptor/neprilysin inhibitor (ARNI)	5 (26.3)	3 (7.7)	6 (17.1)	0.16
ARNI/ACEI/ARB	14 (73.7)	31 (79.5)	25 (71.4)	0.71
Mineralocorticoid receptor antagonist (MRA)	4 (21.1)	12 (30.8)	8 (22.9)	0.64
Sodium-glucose cotransporter-2 (SGLT2) inhibitors	1 (5.3)	1 (2.6)	4 (11,4)	0.3
Loop diuretics	10 (52.6)	21 (53.8)	22 (62.9)	0.67

**Table 4:** Influence of comorbidities on heart failure medication.

Treatment	Comorbidity
Angiotensin-converting enzyme (ACE) inhibitors, Angiotensin II receptor blockers (ARBs), Angiotensin receptor/neprilysin inhibitor (ARNI)	Chronic renal insufficiency n = 20 (21.5%) Hypotension n = 11 (11.8%)
Mineralocorticoid receptor antagonist (MRA)	Chronic renal insufficiency n = 7 (7.5%) Hyperkalemia n = 3 (3.2%)
Beta-blockers	Hypotension n = 10 (10.8%) Bradycardia n = 5 (5.4%)
Sodium-glucose cotransporter-2 (SGLT2) inhibitors	Recurrent urinary tract infections n = 2 (2.2%) Chronic renal insufficiency n = 10 (10.8%)
Other reasons for influence of overall heart failure treatment:	Frailty n = 22 (23.7%) Dementia n = 12 (12.9%) Best supportive care n = 10 (10.8%) Non-compliance n = 5 (5.4%)

## Data Availability

Data are contained within the article.
